# Variability in the Timing of Respiratory Syncytial Virus Epidemics in Guatemala, 2008–2018

**DOI:** 10.1111/irv.13334

**Published:** 2024-07-09

**Authors:** Sarah Hamid, Laura M. Grajeda, Oscar de Leon, Maria Renee Lopez, Herberth Maldonado, Ana Beatriz Gomez, Benjamin Lopman, Thomas F. Clasen, John P. McCracken

**Affiliations:** ^1^ Department of Epidemiology, Rollins School of Public Health Emory University Atlanta Georgia USA; ^2^ Centro de Estudios en Salud Universidad del Valle de Guatemala Guatemala City Guatemala; ^3^ Global Health Institute, College of Public Health University of Georgia Athens Georgia USA; ^4^ Gangarosa Department of Environmental Health, Rollins School of Public Health Emory University Atlanta Georgia USA

**Keywords:** Central America, respiratory syncytial virus infections, respiratory tract diseases, seasons

## Abstract

**Background:**

The description of local seasonality patterns in respiratory syncytial virus (RSV) incidence is important to guide the timing of administration of RSV immunization products.

**Methods:**

We characterized RSV seasonality in Guatemala using the moving epidemic method (MEM) with absolute counts of RSV‐associated acute respiratory infections (ARI) from hospital surveillance in Santa Rosa and Quetzaltenango departments of Guatemala.

**Results:**

From Week 17 of 2008 through Week 16 of 2018, 8487 ARI cases tested positive for RSV by rRT‐PCR. Season onsets varied up to 5 months; early seasons starting in late May to early August and finishing in September to November were most common, but late seasons starting in October to November and finishing in March to April were also observed. Both epidemic patterns had similar durations ranging from 4 to 6 months. Epidemic thresholds (the levels of virus activity that signal the onset and end of a seasonal epidemic) calculated prospectively using previous seasons' data captured between 70% and 99% of annual RSV detections. Onset weeks differed by 2–10 weeks, and offset weeks differed by 2–16 weeks between the two surveillance sites.

**Conclusions:**

Variability in the timing of seasonal RSV epidemics in Guatemala demonstrates the difficulty in precisely predicting the timing of seasonal RSV epidemics based on onset weeks from past seasons and suggests that maximal reduction in RSV disease burden would be achieved through year‐round vaccination and immunoprophylaxis administration to at‐risk infants.

## 1 Introduction

Respiratory syncytial virus (RSV) is the major viral respiratory tract infection of early infancy and the most common cause of hospitalizations in infants globally [1]. It has been estimated to cause about 34 million episodes of acute lower respiratory tract infections in young children each year, with over 3 million severe enough to cause hospitalization [2]. In terms of mortality, an estimated 66,000–199,000 deaths in children under 5 years old are attributed to RSV, the vast majority in developing countries [2].

Until recently, no RSV vaccines were available, and the only licensed monoclonal antibody (mAb) was generally not accessible in low‐resource settings due to its cost and monthly dosing delivery logistics [3, 4, 5]. However, as of late 2022, a vaccine for older adults and pregnant women and a single‐dose long‐acting mAb (nirsevimab) were market approved [6]. Nirsevimab has been shown to be effective through 150 days after injection; thus, it is important to administer it during periods that will provide protection to infants during the RSV season. A maternal vaccine against RSV provides passive immunity of limited duration to infants (maternal antibodies wane by about 6 months), so timing of administration is also important [7].

Characterizing seasonality—that is, cyclical patterns in infection incidence—is important to guide the timing of administration of mAbs and vaccines. Global reviews have served as a guide to RSV seasonality [8]. However, seasonal patterns are often variable between as well as within countries, so characterizing national and local seasonality patterns is needed to inform effective national vaccine strategies and the timing of prevention measures [9]. Thus, the World Health Organization has identified the description of local seasonality patterns in RSV incidence as a priority research activity [3].

RSV seasonality is correlated with geographic location and climate. In temperate regions, RSV activity tends to occur in colder, drier months, and seasonal epidemics last about 5–6 months [10]. Numerous explanations for these patterns have been proposed, including the possibility that inclement weather modifies human behavior, increasing indoor crowding that enhances exposure and transmission of RSV, or that low temperatures and absolute humidity increase the risk of infection [11, 12, 13]. In contrast to temperate areas, in subtropical and tropical climates, peak activity typically occurs during the warmest months, and RSV seasons last longer, up to 10 months [10, 14]. A global overview of RSV seasonality found that most countries with available data on RSV have consistent seasonal patterns, but seasonal parameters can change over time [10]. Although the specific drivers of RSV seasonality remain unclear, identifying seasonal patterns and defining epidemic thresholds (the level of RSV activity that signifies the onset of a seasonal epidemic) are important when planning prevention strategies such as vaccination [7].

Few Central American countries have published research on RSV seasonality, and previous studies relied on only a few years of data, making it difficult to detect long‐term trends. Analyses of hospitalized acute respiratory infections in Guatemala between 2007 and 2012 described temporal patterns of RSV infections using visual inspection of epidemiologic curves [15, 16]. A recent global overview of RSV seasonality included 2 years (2015–2017) of data from Guatemala [10]. However, as patterns in RSV seasonality can shift over time, it is important to characterize seasonality over many years [10].

There is no standard approach to characterizing respiratory virus epidemics. However, for influenza, the moving epidemic method (MEM) has been used to determine epidemic and alert thresholds that respectively signal the onset and severity of seasonal influenza epidemics [17, 18]. The method defines a baseline based on several years of data and establishes an epidemic threshold above which weekly virus activity is in the epidemic period. Although this method was designed primarily for influenza epidemic and pandemic severity assessment, it can be used with any data that have a seasonal accumulation of cases to characterize key aspects of seasonality, including season onsets, offsets, and durations. The MEM has recently been used to describe the seasonality of RSV in the Netherlands and in Slovenia [19, 20].

The objective of this study was to characterize RSV seasonality in Guatemala from 2008 to 2018 using the MEM.

## 2 Methods

### 2.1 Study Setting and Population

The main data source for this analysis is *Vigilancia Integrada Comunitaria* (VICo), an integrated infectious disease surveillance system in Guatemala established through a collaboration between the US Centers for Disease Control and Prevention International Emerging Infections Program, the Guatemala Ministry of Public Health and Welfare, and the Universidad del Valle de Guatemala. The surveillance system has been described previously [15]. Briefly, it was established in November 2007 in Santa Rosa Department and subsequently expanded to Quetzaltenango Department in February 2009. Among other syndromes, VICo includes surveillance of hospitalized acute respiratory tract infections (ARI).

Surveillance for hospitalized ARI in Santa Rosa (eastern Guatemala, with a maximum elevation of 1330 m and temperatures typically ranging from 15°C to 30°C) [21] was conducted at the department's only public hospital, the Cuilapa National Hospital, a regional referral hospital with 176 beds at the start of the study period. In Quetzaltenango (western Guatemala, with a maximum elevation of 2330 m and temperatures ranging from 6°C to 21°C) [21], surveillance was at Western Regional Hospital, which had 435 beds at the start of the study period. Both facilities include pediatric and adult intensive care units. Although the age distributions of the surveillance populations in the two departments are similar, demographic characteristics and health‐seeking behaviors differ. The populations of Santa Rosa and Quetzaltenango are 46% and 62% urban, respectively, and the populations have different ethnic compositions [22]. Healthcare utilization surveys carried out in Santa Rosa and Quetzaltenango in 2007 and 2009, respectively, found that among those reporting a hospitalization for pneumonia in the past year, 33% of those aged < 5 years and 75% of those aged ≥ 5 years in Santa Rosa were admitted to the surveillance hospital, whereas in Quetzaltenango, 75% of those aged < 5 years and 50% of those aged ≥ 5 years were admitted to the surveillance hospital [15, 23].

### 2.2 Case Definitions and Study Procedures

A case of ARI was defined as a hospitalization with at least one sign or symptom of respiratory disease and evidence of acute infection within the first 24 h of admission (Table 1). Study nurses screened the ward registers and emergency department logs for patients presenting with respiratory signs and symptoms. Study staff assessed eligibility based on chart review and patient interview and sought consent and enrollment of hospitalized patients meeting the ARI case definition. All participants were asked to provide nasopharyngeal and oropharyngeal swabs. Nurses collected samples and placed them in viral transport media, combining the swabs in a single vial. Specimens were tested by rRT‐PCR for a respiratory panel that includes RSV at Universidad del Valle de Guatemala laboratory using protocols provided by the Centers for Disease Control and Prevention. Samples were processed within 72 h of collection.

**TABLE 1:** Surveillance case definitions for hospitalized acute respiratory infection in Guatemala.

Evidence of acute infection^a^	Signs or symptoms of respiratory disease^a^	
All ages	All ages	Criteria for children < 2 years
≥ 1 of the following:	≥ 1 of the following:	≥ 1 of the following:
Fever (≥ 38°C) or history of fever	Tachypnea	Repeated pauses in breathing while breastfeeding or drinking
Hypothermia (< 35.5°C)	Cough	Intercostal retractions
Abnormal white blood cell count	Expectoration (sputum production)	Nasal flaring
Abnormal white blood cell differential	Chest pain	Grunting
	Hemoptysis	Not drinking or breastfeeding
	Dyspnea (difficulty breathing)	
	Shortness of breath	
	Sore throat	
	Abnormal lung examination	

*Note:* Tachypnea was defined as age < 2 months: ≥ 60 breaths/min, age 2–12 months: ≥ 50 breaths/min, age > 12 months to 5 years: ≥ 40 breaths/min, age > 5 years: ≥ 20 breaths/min. Abnormal white blood cell count (in children < 5 years: < 5500/cm^3^ or > 15,000 / cm^3^, in people ≥ 5 years: < 3.000/cm^3^ or > 11,000/cm^3^). Any white blood cell differential abnormality as defined by the automated blood cell analyzer at each surveillance site.

^a^ Only patients with onset of signs or symptoms in the past 14 days met the case definition. Children aged < 2 years who met the criteria for all ages or the criteria for children < 2 years old were considered ARI cases.

### 2.3 Study Data and Season Attributes

To describe RSV seasonality in Guatemala, we used the absolute numbers of RSV detections from ARI surveillance in Santa Rosa and Quetzaltenango from Week 17 of 2008 to Week 16 of 2018. Data for the 2008–2009 season were available for Santa Rosa but not for Quetzaltenango, so pooled analyses were restricted to data collected from Week 17 of 2009 through Week 16 of 2018.

We used the MEM to define attributes of RSV seasonality including the onset (start) week, peak week, time from onset to peak week, offset (end) week, epidemic duration, percentage of annual RSV detections that occur within the epidemic period, and the pre‐ and post‐epidemic thresholds (i.e., the number of RSV detections that respectively mark the start and end of the seasonal epidemic). The MEM is described elsewhere and was applied using the “mem” R package version 2.17 and MEM Shiny Web Application with R Version 4.2.2 [18, 24]. Briefly, the MEM is a mathematical algorithm that divides each surveillance season (defined as Week 17 to Week 16 based on typical troughs in RSV activity in Guatemala) separately into pre‐epidemic, epidemic, and post‐epidemic weeks based on the slope of the epidemic curve. A pre‐epidemic threshold that marks the start of the epidemic is calculated using a set of pre‐epidemic counts of weekly RSV detections from past seasons. For each season, the highest *n* weekly detections from the pre‐epidemic period are taken, where *n* = 30/number of seasons. The pre‐epidemic threshold is the upper bound of the one‐tailed 95% confidence interval of the geometric mean of this subset of the highest 30 pre‐epidemic weekly counts of all seasons. A post‐epidemic threshold that marks the end of the epidemic is calculated by repeating this process using post‐epidemic counts.

Differences in RSV seasonality in Santa Rosa and Quetzaltenango were assessed by running the MEM algorithm on site‐stratified data to calculate the season parameters described above. We also calculated Spearman's rank order correlation coefficient to test whether weekly RSV counts in the two sites were positively correlated.

### 2.4 Assessing Threshold Performance

To assess how well the MEM epidemic thresholds would have performed prospectively, we calculated the proportion of annual RSV detections that occurred within forecasted seasonal epidemics (defined based on pre‐epidemic and post‐epidemic thresholds calculated from previous seasons' data). The 2012/2013 epidemic thresholds were calculated using data from 2009/2010 through 2011/2012, and the 2013/2014 thresholds were calculated using data from 2009/2010 through 2012/2013, and so on. We did not calculate thresholds for the 2009/2010 to 2011/2012 seasons as there were too few previous seasons of data available. We considered the forecasted onset and offset weeks of each seasonal epidemic to be the first and last of 2 consecutive weeks above the pre‐ and post‐epidemic thresholds, respectively. Assuming one RSV epidemic wave per surveillance season, we defined the epidemic period as the longest period of weekly counts above the epidemic thresholds. False alerts were defined as weeks outside the epidemic period that had RSV counts above the epidemic thresholds.

### 2.5 Assessing Differences in Seasonality by Age

Variability of age distributions among persons hospitalized with RSV over the nine consecutive seasons was assessed using proportional stacked bar graphs; age was categorized into six groups: 0–5 months, 6–11 months, 1–2 years, > 2–5 years, > 5–65 years, and > 65 years. A chi‐square test was used to assess differences in age groups across seasons. Additionally, the Kruskal–Wallis test was used to test whether the continuous age distributions of RSV hospitalizations differed across seasons.

## 3 Results

**FIGURE 1:**
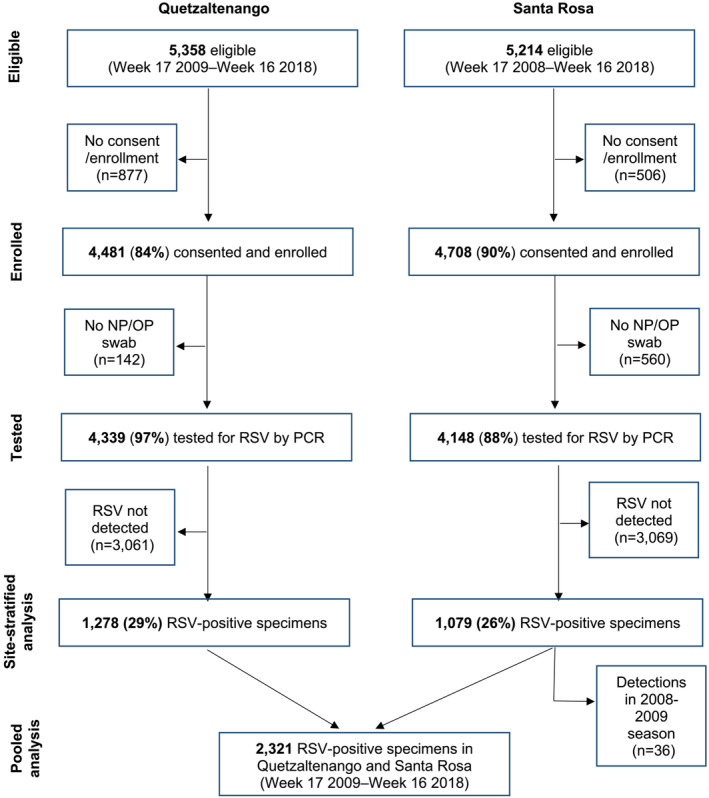
Selection of RSV hospitalizations for analytic data set from hospitalized surveillance in Guatemala, 2008–2018.

From Week 17 of 2008 through Week 16 of 2018, 8487 ARI cases were enrolled and tested for RSV at Cuilapa National Hospital in Santa Rosa (*n* = 4148) and Western Regional Hospital in Quetzaltenango (*n* = 4339) (Figure 1). Analyses pooled across the two sites excluded data from 2008 and were restricted to 8222 ARI cases enrolled and tested for RSV from Week 17 of 2009 through Week 16 of 2018. During this period, 1043 (27%) and 1278 (29%) ARI cases tested positive for RSV by PCR at Cuilapa National Hospital and Western Regional Hospital, respectively. The median number of RSV detections per season was 223 (IQR: 198, 330). The majority (70%, *n* = 1618) of cases were in children less than 1 year of age. Adults aged ≥ 65 years comprised 2% (*n* = 54) of cases.

**FIGURE 2:**
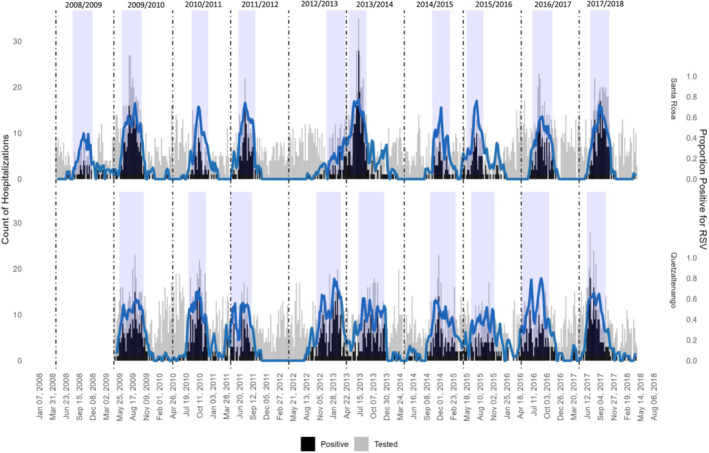
Number of PCR tests for RSV, RSV positive tests, and proportion positive among acute respiratory infection cases by surveillance site, Guatemala, April 2008–April 2018 (*n* = 8487). Figure displays weekly numbers of acute respiratory infections (ARI) tested by PCR for RSV (a), positive for RSV (b), and the proportion positive for RSV (b/a). Data are from two hospital surveillance sites: one in Santa Rosa and another in Quetzaltenango from Week 17 of 2008 to Week 16 of 2018. Surveillance in Quetzaltenango began in February 2009 and lack data for the 2008–2009 season. Vertical dotted lines mark surveillance seasons (defined as epidemiologic weeks 17 through 16). The solid blue line represents a 3‐week centered moving average of the proportion of ARI cases positive for RSV. The shaded blue regions indicate the seasonal epidemic period as defined by the moving epidemic method.

RSV seasons varied substantially by year such that two patterns of seasonality were observed: an early season starting in May to early August and a late season starting in October/November (Figure 2). Epidemic onsets ranged from Week 20 in the 2011/2012 season to Week 44 in the 2012/2013 season (Table 2). We did not observe a consistent pattern of late seasons oscillating with early seasons or small seasons oscillating with larger ones. The median peak week of the epidemic occurred at Week 33 (range: 28–9). Offset weeks ranged from Week 37 of 2011 in the 2011/2012 season to Week 16 of 2013 in the 2012/2013 season. The epidemic duration ranged from 17 to 24 weeks and included 74%–95% of annual RSV detections.

**TABLE 2:** Characteristics of respiratory syncytial virus (RSV) epidemics using the moving epidemic method (MEM) in Guatemala, April 2009–April 2018.

A. Surveillance season	B. Onset week	C. Peak week	D. Offset week	E. Epidemic duration, weeks	F. RSV detections in the epidemic period (%)	G. Time from onset to peak, weeks (no.)	H. Pre‐epidemic threshold, RSV detections/week	I. Post‐epidemic threshold, RSV detections/week	J. Forecasted epidemic duration, weeks (no.)	K. RSV detections in forecasted epidemic period (%)	L. False alert, (no.)
2009/2010	22	35	42	21	90%	13					
2010/2011	31	41	47	17	84%	10					
2011/2012	20	30	37	18	90%	10					
2012/2013	44	9	16	24	92%	17	1.8	2.0	32	99%	0
2013/2014	23	28	43	21	74%	5	2.3	2.4	36	98%	0
2014/2015	43	48	11	21	84%	5	3.1	4.1	15	70%	1
2015/2016	24	29	45	22	76%	5	3.1	3.9	21	75%	1
2016/2017	25	33	46	22	83%	8	3.4	4.3	26	91%	0
2017/2018	24	30	44	21	95%	6	3.6	4.0	21	95%	0
Median	24	33	46	21	84%	8	3.1	4.0	23.5	93%	0

*Note:* B–G represent values calculated using the MEM algorithm for each season separately. Epidemic thresholds (H,I) for each target season (A) were calculated using data from previous seasons, starting with the 2009/2010 season. Epidemic duration (D) is the number of weeks between epidemic onset (B) and offset (D) weeks inclusive. Forecasted epidemic duration (J) is calculated using onset and offset weeks defined as the first and last of 2 consecutive weeks above the pre‐ and post‐epidemic thresholds, respectively. The percentage of RSV detections in the forecasted epidemic period (K) is defined as the proportion of annual RSV detections that occurred within the forecasted seasons (i.e., between pre‐ and post‐epidemic thresholds). False alerts (L) were defined as weeks outside the forecasted epidemic period that had RSV counts above the epidemic thresholds.

A median of six (IQR: 2–11) RSV detections were reported each week. Using data from all surveillance seasons, the number of RSV detections that marked the start of the seasonal epidemic (pre‐epidemic threshold) was 3.7 cases/week, and the post‐epidemic threshold was 4.0 cases/week. Forecasted MEM pre‐epidemic thresholds ranged from 1.8 to 3.6 RSV hospitalizations/week, and post‐epidemic thresholds ranged from 2.0 to 4.3 RSV hospitalizations/week. Between 70% and 99% of annual RSV detections occurred above the epidemic thresholds (Table 2). The number of weeks in a given surveillance season that were above the epidemic threshold ranged from 15 to 36. The MEM thresholds produced a false positive alert in two seasons.

Although there was a moderate positive relationship between weekly counts of RSV hospitalizations in Santa Rosa and Quetzaltenango (Spearman's rank order correlation coefficient = 0.63, *p* < 0.01), the seasons in the two sites diverged somewhat (Figure 2). Across the seasons, onset weeks in the two sites differed by 2–10 weeks each season, and offset weeks differed by 2–16 weeks (Table 3). In most seasons, epidemic onsets occurred earlier (seven of nine seasons) and ended earlier (five of nine seasons) in Quetzaltenango. However, there were exceptions to this pattern. For example, the 2013/2014 season started, peaked, and ended about 2 months earlier in Santa Rosa than in Quetzaltenango.

**TABLE 3:** Characteristics of respiratory syncytial virus (RSV) epidemics, by surveillance site, using the moving epidemic method, Guatemala, April 2008–April 2018.

	Onset week		Peak week		Offset week		Epidemic duration, weeks		Percentage of RSV detections in the epidemic period		Time from onset to peak, weeks	
	SR	QU	SR	QU	SR	QU	SR	QU	SR	QU	SR	QU
2008/2009	32	—	42	—	49	—	18	—	72%	—	9	—
2009/2010	23	21	30	35	41	43	19	23	93%	89%	7	14
2010/2011	34	31	40	41	49	47	16	17	90%	82%	6	10
2011/2012	24	17	30	27	39	36	16	20	93%	91%	6	10
2012/2013	51	42	11	9	16	12	18	23	81%	89%	12	19
2013/2014	19	28	28	45	35	51	17	24	85%	86%	9	17
2014/2015	42	40	49	48	5	10	17	24	88%	84%	7	8
2015/2016	20	24	29	39	35	45	16	22	76%	75%	9	15
2016/2017	27	17	34	25	45	42	19	26	94%	88%	7	8
2017/2018	27	24	36	27	44	41	18	18	97%	88%	9	3

Abbreviations: QU, Quetzaltenango; SR, Santa Rosa.

**FIGURE 3:**
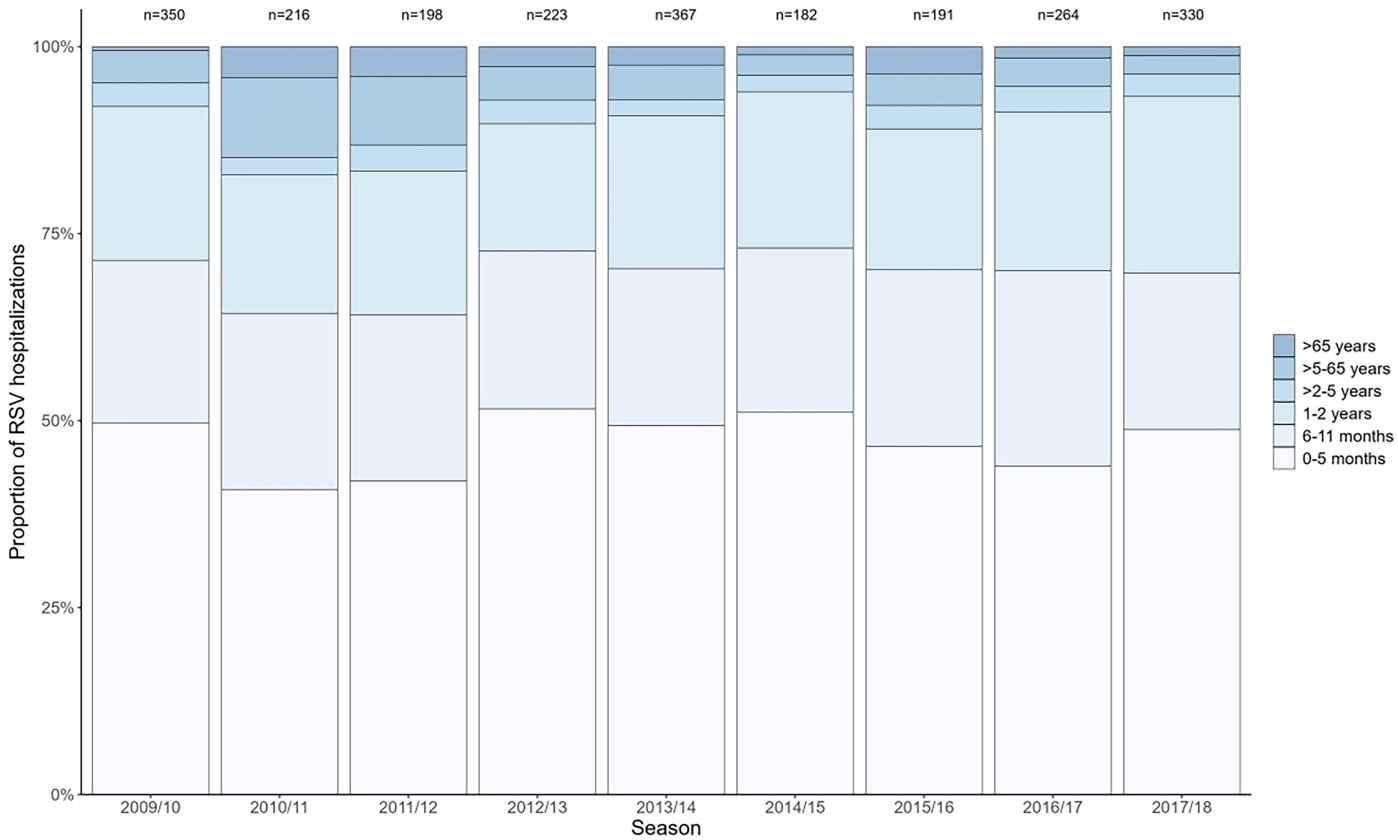
Distribution of hospitalized patients with respiratory syncytial virus (RSV) infections in nine consecutive seasons, according to age group, Guatemala, 2009/2010–2017/2018 (*n* = 2321 RSV‐associated ARI cases). N.B. The 2009/2010–2011/2012, 2013/2014, and 2015/2016–2017/2018 epidemics began in late May to early August (early onset), whereas the 2012/2013 and 2014/2015 epidemics began in October/November (late onset).

Although inter‐season differences in the proportions of RSV hospitalizations in each age group were statistically significant (chi‐square *p* value = 0.03), there was no statistical evidence of a difference in continuous age distributions across seasons (Kruskal–Wallis *p* value = 0.08). The highest proportion of RSV hospitalizations was consistently in the ages < 6 months (range: 45%–57%), and the lowest proportions of RSV hospitalizations were in the age groups ≥ 65 years (range: 1%–4%) and > 2–5 years (range: 2%–4%) (Figure 3). In both early (2009/2010–2011/2012, 2013/2014, 2015/2015–2017/2018) and delayed (2012/2013 and 2014/2015) seasons, the median age of children < 5 years old hospitalized with RSV was 5 months.

## 4 Discussion

Our analysis of data from prospective surveillance of hospitalized ARI over 10 consecutive seasons provides a detailed overview of seasonal patterns in RSV hospitalizations in Guatemala. Our results demonstrate substantial variability in the timing of RSV epidemics. Epidemic onsets varied by as much as 5 months such that two patterns of RSV seasonality were observed: an early season starting in May to early August and finishing in September to November and a late season starting in October to November and finishing in March to April, with both seasons having similar durations ranging from 4 to 6 months. This variability demonstrates the difficulty in precisely predicting the timing of seasonal RSV epidemics based on onset weeks from past seasons.

To be useful, thresholds should capture a high proportion of annual RSV detections in a short period of time. MEM epidemic thresholds calculated separately for each season from previous seasons' data gave an indication of how well the MEM epidemic threshold would forecast epidemic onsets and offsets. Thresholds calculated in this way performed well in terms of the proportion of annual RSV detections in the forecasted epidemic period. In each season, at least 70% of annual RSV detections occurred in weeks above the epidemic thresholds. Typically, about 5–6 months were above the epidemic threshold, but the epidemic period was particularly long (8–9 months) in the 2013/2014 season, perhaps due to the staggered timing of regional epidemics during that season.

We hypothesized that climatic differences between Santa Rosa (coastal lowlands) and Quetzaltenango (western highlands) might result in differing seasonal patterns. Elsewhere, including the United States, there is substantial subnational variation in RSV seasonality, with the RSV season starting in the southeast and then moving to the northwest [9]. In Guatemala, seasonal epidemics typically started earlier in Quetzaltenango, but the drivers of this pattern are unclear. Stratifying the analysis by site revealed important patterns that were masked in the pooled data. For example, the 2012/2013 and 2013/2014 seasons appear to be one prolonged season in the pooled data, but stratification shows a distinct late epidemic followed by an early epidemic in Quetzaltenango that is masked by a delayed 2012/2013 season that peaked in early 2013 in Santa Rosa. As has been noted elsewhere, this demonstrates that pooling national data may misrepresent RSV activity and that local data are needed to precisely define RSV outbreaks in a given community [25].

We had hypothesized that the age distribution of RSV cases might differ across seasons, with cases being older on average in seasons with later onsets. Elsewhere, delayed or off‐season RSV epidemics have been associated with increased age of RSV cases due to the accumulation of RSV‐naïve children, notably during the COVID‐19 pandemic [26]. There was little inter‐season variability in age distribution of RSV cases, and we did not find differences in the median age of children with RSV in delayed seasons and early seasons. This may be because delays in season onset were not of sufficient magnitude to result in shifted age distributions.

The occurrence of distinct seasonal epidemics with a relatively stable duration in Guatemala was consistent with RSV patterns in other areas [8]. However, epidemics in most countries are consistent over time with year‐to‐year variations of 1–4 weeks in the start, end, and/or peak of RSV activity, whereas we observed greater variability across years. Similar multiyear periodicity has been reported in a few countries [10, 27]. For example, in Mexico, a year with two epidemics is followed by a milder year, where the outbreak starts in spring and activity is maintained almost all year round with no clear peaks [10].

The mechanisms that shape these seasonal RSV patterns are unclear but include contact rates between susceptible and infected individuals and host immunity. Although meteorological factors have been found to predict RSV incidence, the correlations between RSV incidence, temperature, and relative humidity are particularly variable and inconsistent in tropical regions [28]. Moreover, mechanistic models have shown that undetectable seasonal changes in transmission can combine with population immunity to produce large oscillations in disease incidence [29]. Although there is limited research on the impact of RSV subtype on RSV seasonality, the predominant circulating antigenic group might also play a role in shaping seasonal patterns. In Finland, RSV antigenic groups A and B alternate in 2‐year cycles, and in South Korea, different genotypes dominate the circulation in consecutive epidemics [30, 31]. A study in Beijing found that longer and earlier epidemics occurred during RSV A dominant seasons [32]. Finally, viral interference can also affect seasonality as several respiratory viruses can circulate during the same period; elsewhere, RSV has been shown to be less frequently detected during influenza epidemics [33].

The observed variability in RSV seasonality has important implications for future vaccine trials and immunization programs. If trials occur during seasons with relatively low RSV activity, they may not achieve adequate power to detect hypothesized effect sizes. Notably, a recent trial fell short of its projected number of RSV‐associated, medically attended lower respiratory tract infections and failed to meet the prespecified criterion for success [34]. Future RSV vaccine trials should span several seasons to ensure that they achieve the targeted number of endpoints. In terms of an eventual immunization strategy, the variability in seasonality in Guatemala suggests that seasonal administration of monoclonal antibodies or maternal vaccination could fail to protect a large proportion of at‐risk infants and young children.

Our study has several limitations and simplifying assumptions that bear noting. Although our pooled analysis included nine seasons of consistently collected surveillance data—more than previous reports of RSV activity in Guatemala—the study period was not of optimal duration for assessing longer‐term trends and multiyear seasonality. We did not have data on RSV subtypes, which have been shown to correlate with different seasonality patterns in some settings [32]. Although surveillance hospitals attempted to test all ARI cases for RSV, this was not always possible. However, it is unlikely that testing practices meaningfully influenced the observed seasonal patterns because a high proportion of ARI cases were tested for RSV (92%) and seasonal patterns in RSV test positivity mirrored those of RSV detections. Finally, we assumed that healthcare‐seeking behavior and the population under surveillance remained stable over the study period; unknown violations of this assumption would have affected our estimates.

Despite these limitations, our study provided a detailed description of RSV seasonality in Guatemala that can guide the timing of prevention strategies such as vaccination and immunoprophylaxis. Characterizing RSV seasonality is important for decision‐making about the timing and strategy for immunization product administration, whereas setting epidemic thresholds that can be used prospectively to signal the start of a seasonal RSV epidemic can improve both the accuracy of clinical diagnosis and the timely use of costly immunoprophylaxis. The inclusion of nine seasons of consistently collected surveillance data allowed for the identification of two differential patterns of seasonality, which was not possible in previous studies that covered shorter time periods. Our findings can provide baseline information for immunization advisory groups and others seeking to assess the effects of RSV vaccines for pregnant women and long‐lasting monoclonal antibodies for infants and young children.

## Author Contributions

**Sarah Hamid:** conceptualization, formal analysis, methodology, writing–original draft. **Laura M. Grajeda:** data curation, investigation, writing–review and editing. **Oscar de Leon:** data curation, investigation, writing–review and editing. **Maria Renee Lopez:** data curation, investigation, writing–review and editing. **Herberth Maldonado:** data curation, investigation, writing–review and editing. **Ana Beatriz Gomez:** investigation, project administration, writing–review and editing. **Benjamin Lopman:** supervision, writing–review and editing. **Thomas F. Clasen:** supervision, writing–review and editing. **John P. McCracken:** conceptualization, funding acquisition, methodology, project administration, supervision, writing–review and editing.

## Conflicts of Interest

BL reports personal fees from Epidemiologic Research and Methods, LLC, and from Hillevax Inc.

## Data Availability

The data that support the findings of this study are available upon reasonable request from the corresponding author. Data access requests will be reviewed by the data access committee at Universidad del Valle de Guatemala.
